# Virtual Screening of Kelch-like ECH-Associated Protein 1-Nuclear Factor Erythroid 2-Related Factor 2 (Keap1-Nrf2) Inhibitors and In Vitro Validation

**DOI:** 10.3390/molecules30081815

**Published:** 2025-04-17

**Authors:** Zhengwan Huang, Zhengang Peng, Dandan Huang, Zhongyu Zhou

**Affiliations:** 1 Bloomage Biotechnology Corporation Limited, Shanghai 200100, China; huangzw@bloomagebiotech.com (Z.H.); huangdand@bloomagebiotech.com (D.H.); 2 Guangdong Provincial Key Laboratory of Applied Botany & Key Laboratory of National Forestry and Grassland Administration on Plant Conservation and Utilization in Southern China, South China Botanical Garden, Guangzhou 510650, China

**Keywords:** Keap1-Nrf2, molecular docking, anti-aging, virtual screening

## Abstract

The transcription factor erythroid 2-related factor 2 (Nrf2) is a central regulator of cellular defense mechanisms against oxidative stress and inflammation. Keap1 (Kelch-like ECH-associated protein 1) regulates Nrf2 activity by ubiquitination-mediated cytoplasmic retention, thereby suppressing its nuclear translocation and subsequent transcriptional activation of genes encoding phase II detoxifying enzymes. Using a structure-based virtual screening approach, we screened ~16,000 natural compounds to identify Keap1-Nrf2 PPI inhibitors. Nine compounds were identified based on their high binding affinities and favorable interactions with Keap1, primarily through non-covalent interactions. To validate the binding stability of these inhibitors, molecular dynamics (MD) simulations were performed, confirming the robustness of the Keap1–inhibitor complexes over time. Subsequent in vitro assays on human epithelial keratinocyte cells (HaCaT) revealed that six of these compounds notably upregulated Nrf2 mRNA expression, regis tering increases from 23% to 50% in comparison to the control. Notably, chebulinic acid emerged as the most potent compound, demonstrating the greatest elevation in Nrf2 expression. Penetration studies further showed that chebulinic acid, when encapsulated in silk fibroin, achieved a 0.14% penetration rate after 24 h though it could not penetrate into the stratum corneum alone. This result highlighted the potential of chebulinic acid in the use of anti-aging skincare formulations. Collectively, our findings affirmed that molecular docking is a reliable and effective approach for the identification of novel anti-aging agents targeting the Keap1-Nrf2 pathway.

## 1. Introduction

Ultraviolet B (UVB, 280–320 nm) induces reactive oxygen species (ROS) generation, including ·O_2_^−^, ·OH, H_2_O_2_, and ^1^O_2_, which drive oxidative cellular damage [1,2]. ROS overaccumulation directly induces covalent damage to critical biomolecules, including DNA, lipids, and proteins [3,4]. Furthermore, ROS contribute to the degradation of collagen by inducing the upregulation of matrix metalloproteinase (MMP) activity, which breaks down the extracellular matrix and impairs skin integrity and elasticity [5]. Both the chemical oxidation of cellular components and the activation of cellular machinery, brought about by ROS, act in concert to cause aging [6,7]. The transcription factor nuclear factor erythroid 2-related factor 2 (Nrf2) promotes the production of approximately 250 antioxidant proteins, including heme oxygenase-1 (HO-1), NAD(P)H quinone oxidoreductase 1 (NQO1), superoxide dismutase (SOD), glutathione S-transferase (GST), and thioredoxin reductase (TRx). It is regarded as a good solution to reduce reactive oxygen species (ROS) production and ROS-related inflammation [8].

The human Nrf2 protein is composed of 605 amino acids and features seven conserved functional domains (Neh1–7). The four proteins, Kelch-like ECH-associated protein 1 (Keap1, also called KLHL19) (which binds to the Neh2 domain of Nrf2), WD repeat domain 23 (WDR23, also called DCAF11), β-transducin repeat-containing protein (β-TrCP) (which bind to the Neh6 domain of Nrf2), and HMG-CoA degradation 1 (HRD1) (which binds to the Neh4–5 domains of Nrf2), trigger the ubiquitination and subsequent degradation of Nrf2 [9]. Keap1 is a dimeric cytoplasmic protein. Human Keap1 comprises 624 amino acids and contains the Broad complex, Tramtrack, and Bric-à-Brac (BTB) domain (for dimerization, recruiting cullin 3 (Cul3), and interacting with Neh2); the BTB and C-terminal Kelch (BACK) domain; the six-bladed β-propeller Kelch-repeat domain (K1-K6), a region represented by ‘+’; and the C-terminal ’C’ domain [10].

Under basal conditions, the Neh2 domain of Nrf2 binds two Keap1 molecules, which homodimerize via their BTB domains. This Keap1 dimer simultaneously recruits the Cullin3 (CUL3)–RING-box 1 (RBX1) E3 ubiquitin ligase complex through its BTB domain, positioning Nrf2 for ubiquitination and proteasomal degradation. Consequently, Nrf2 levels remain tightly regulated post-translationally via this ubiquitin–proteasome system (UPS) [11]. Upon exposure to oxidative or xenobiotic stress, the Keap1-Nrf2 interaction is disrupted, blocking Nrf2 ubiquitination. Stabilized Nrf2 translocates to the nucleus, where it dimerizes with a small musculoaponeurotic fibrosarcoma (sMAF) protein. This heterodimer activates the transcription of antioxidant/anti-inflammatory genes by binding to antioxidant response elements (AREs) in their promoters. The Nrf2–small Maf heterodimer binds to antioxidant response elements (AREs) within the promoter regions of cytoprotective genes, initiating the transcriptional activation of ARE-driven genes, such as heme oxygenase-1 (HO-1), NAD(P)H quinone oxidoreductase 1 (NQO1), and anti-inflammatory mediators. This coordinated upregulation enhances cellular defense mechanisms against oxidative stress and inflammation. The dual antioxidant and anti-inflammatory properties of the Nrf2–ARE pathway are recognized as key molecular targets for anti-aging interventions, offering therapeutic potential in age-related pathologies characterized by redox imbalance and chronic inflammation [12].

The Keap1 protein is the well-studied and most critical Nrf2 negative regulator, targeted by both covalent and non-covalent strategies. The covalent strategy, through the modification of reactive cysteines, has been clinically validated as a reasonable approach [13], with examples including dimethyl fumarate and sulforaphane [13,14]. Nevertheless, these compounds may modify off-target cysteines in the proteome, leading to potential toxicity. Consequently, there is growing interest in non-covalent strategies that directly inhibit the Keap1-Nrf2 protein–protein interaction (PPI). Such inhibitors are expected to offer higher target selectivity and improved safety, compared to covalent inhibitors [15]. Wang et al. screened timosaponin B II from a natural product library of 4533 compounds as a Keap1-Nrf2 inhibitor [16]. Zihao Chen screened 1671 natural compounds through molecular docking and identified KCB-F06 as a Keap1-Nrf2 PPI inhibitor with high binding affinity for the Keap1 Kelch domain, demonstrating its potential to disrupt this interaction [17].

Natural products and traditional Chinese medicine are widely utilized in the treatment of various illnesses, as documented in pharmacopoeias. Natural compounds like curcumin [18] and lycopene [19] have been identified as potent Nrf2 agonists. This suggests that natural Nrf2 agonists may be employed as chemopreventive and chemotherapeutic agents for anti-aging applications in skincare products.

Virtual screening is a computer-aided drug discovery (CADD) method used to identify potential candidates from a silicon database. Compared to in vitro screening, virtual screening significantly reduces both costs and time. The methods are broadly categorized into structure-based and ligand-based approaches, differentiated by the molecular information used for model construction: target protein structures or pharmacophore profiles of known bioactive molecules, respectively [20,21]. This study focuses on computational ligand prioritization using Keap1-Nrf2 interaction modeling, with mRNA-level screening serving as the primary functional filter—a well-established strategy in natural product active discovery [22].

## 2. Results and Discussion

### 2.1. Molecular Docking Screening Model Construction

Seven Keap1 commercial inhibitors from the ChEMBL database were selected to establish the molecular docking screening model of Keap1 inhibition. These seven inhibitors were listed in the database as ChEMBL3601212, ChEMBL3899754, ChEMBL4174651, ChEMBL4544116, ChEMBL4646536, ChEMBL4757197, and ChEMBL4762197, which showed half-maximal inhibitory concentration values (IC_50_) ranging from 0.14 to 120 nmol/L.

The docking results (Table 1) showed that all seven inhibitors had good binding affinity with Keap1 (with binding energies below −9 kcal/mol, indicating strong binding interactions [23]).

**Figure 1:**
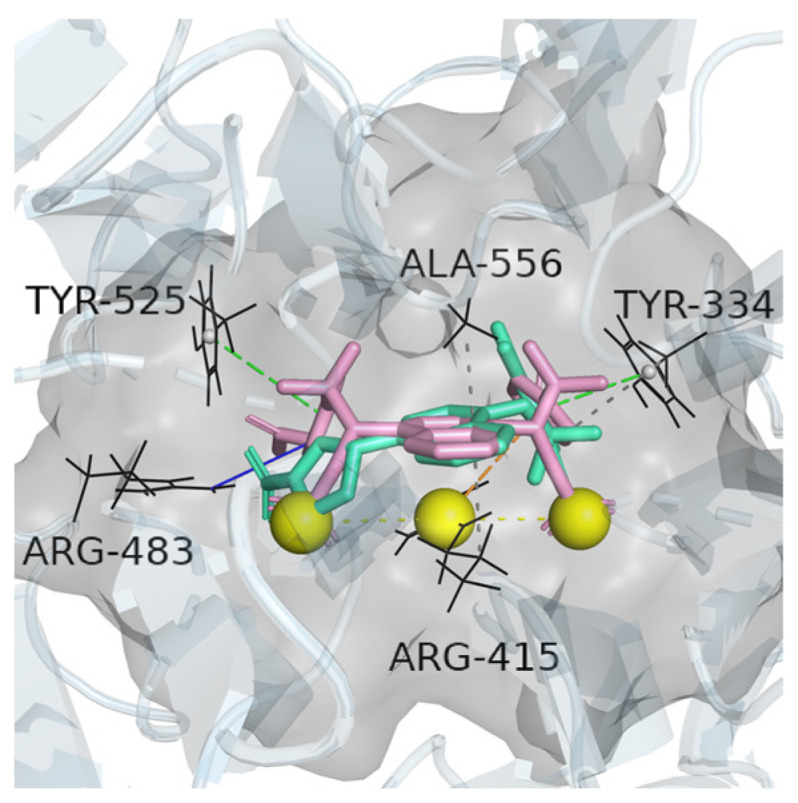
Binding of ChEMBL3899754 (green) and ChEMBL3601212 (purple) into the active pocket (gray) of Keap1.

From Table 1, Tyr334, Arg483, Arg415, Tyr525, and Ala556 were identified as the most frequently occurring key residues in Keap1 that interacted with seven inhibitors. These inhibitors competitively bound to these residues, thereby promoting Nrf2–Keap1 dissociation, a finding consistent with Xing Luo’s study [24]. The interactions of ChEMBL3899754 and ChEMBL3601212 with specific residues in the active pocket of Keap1 are presented in Figure 1. The interaction analysis by PLIP (Protein–Ligand Interaction Profiler) showed that the inhibitor interacted with the residuals of Keap1 in the forms of hydrogen bonds, pi–cation interactions, and hydrophobic interactions, which indicated that the interactions between inhibitors and Keap1 were non-covalent rather than covalent with the Cys151 of Keap1 [25]. Based on the established model, we proposed that the Keap1 inhibitors selected for virtual screening must meet two criteria: First, the binding energy should be less than −9 kcal/mol. Second, the interacting residues with Keap1 must include at least four of the following five amino acid residues: Tyr334, Arg483, Arg415, Tyr525, and Ala556. Furthermore, considering the research and development costs, the price of the virtual Keap1 inhibitors should be limited to 400 yuan per milligram.

### 2.2. Virtual Screening of Keap1 Inhibitors

**Figure 2:**
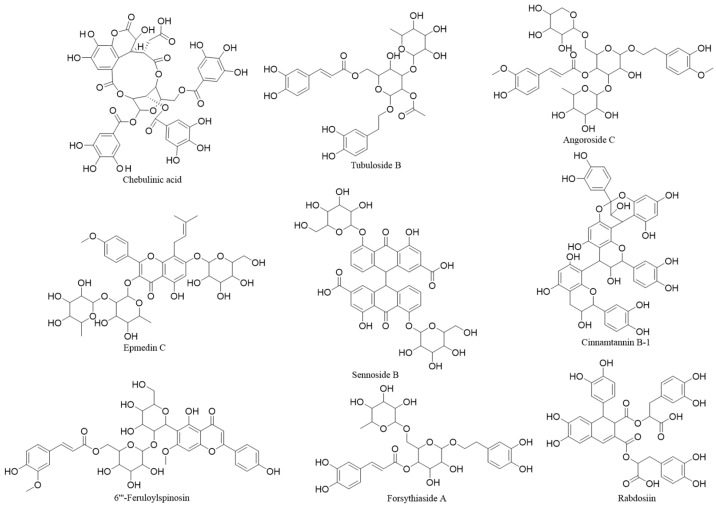
Nine Keap1 inhibitors identified by virtual screening.

**Figure 3:**
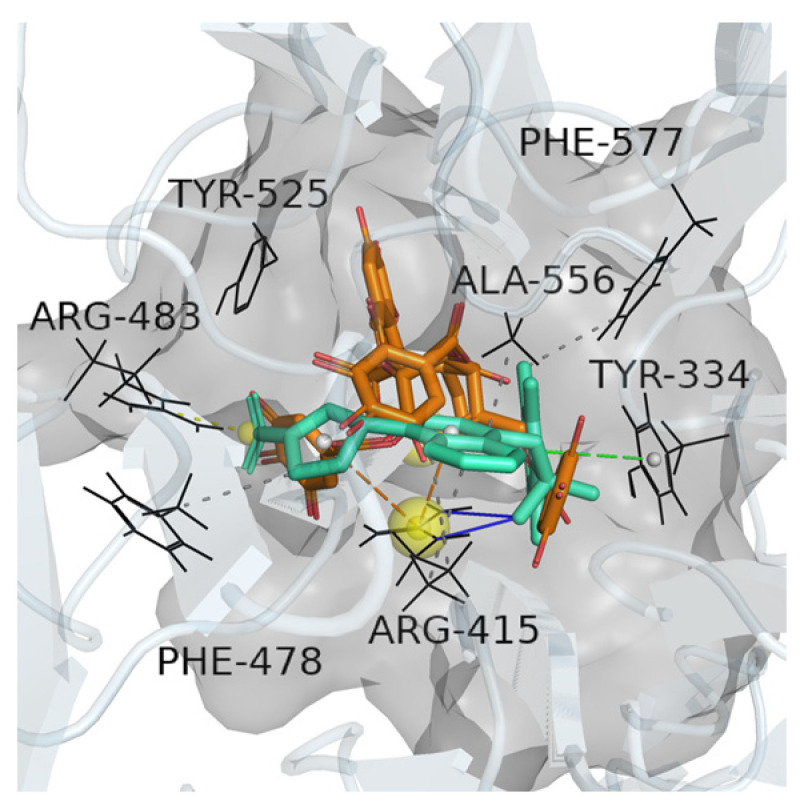
Binding of ChEMBL3899754 (green) and chebulinic acid (orange) into the active pocket (gray) of Keap1.

Our investigation evaluated around 16,000 natural compounds (Supplementary Information, Table S1 summarizes the compounds for virtual screening and their binding energies with the Keap1 protein) for their potential as Keap1 inhibitors, with a focus on disrupting the Keap1-Nrf2 protein–protein interaction (PPI). Based on the virtual screening model, nine compounds were identified as candidate inhibitors, demonstrating high binding energy and interactions with ≥4 key amino acid residues in the target protein. The nine compounds were chebulinic acid, tubuloside B, angoroside C, epmedin C, sennoside B, cinnamtannin B-1, 6‴-feruloylspinosin, forsythiaside A, and rabdosiin. The corresponding molecular architectures are presented in Figure 2 The binding energy and interacted amino acid residuals (see Supplementary Information, Figure S1) are recorded in Table 2. The specific interactions between the chebulinic acid and Keap1 are illustrated in Figure 3.

From Table 2, the candidate inhibitors were observed to bind to key residues in Keap1 primarily through hydrogen bonding and hydrophobic interactions. As shown in Figure 3, all nine inhibitors featured multiple hydroxyl and carbonyl groups, which facilitated hydrogen bonding with Keap1 residues, as well as benzene rings that contributed to hydrophobic interactions. These findings align with prior studies [26,27], which highlight hydrophobic and hydrogen bonding interactions as critical driving forces in the association between phenolic compounds and proteins. Furthermore, the calculated binding energies of certain inhibitors were comparable to those of commercial inhibitors, suggesting that these candidate inhibitors may also exhibit strong inhibitory potential.

### 2.3. ADMET Study for the Predicted Compounds 

Docking analyses demonstrated that the candidate compounds bound effectively to Keap1, confirming their potential as inhibitors targeting Keap1. According to the online server “https://biosig.lab.uq.edu.au/pkcsm (accessed on 25 March 2025)”, these inhibitors were further evaluated (see Supplementary Information, Table S2) in terms of ADMET. The data revealed that the molecular weight (Mw) of the inhibitors ranged from 624–956 Da. Based on Lipinski’s Rule of Five, the nine compounds could have low or medium intestinal absorption, which meant they could also have a low permeation of stratum corneum. However, these challenges could be addressed by integrating novel delivery strategies, such as nanocarriers, advanced transdermal devices, or permeation enhancers, as suggested in a prior work [28]. For instance, retinyl palmitate-loaded solid lipid nanoparticles (SLNs) have demonstrated enhanced skin protection and penetration in anti-wrinkle applications [29], highlighting the viability of such approaches. Thus, designing a tailored delivery system for these inhibitors is essential to overcome their pharmacokinetic limitations. For the toxicity, all the compounds showed no hepatotoxicity and skin sensitization.

### 2.4. Molecular Dynamics (MD) Simulations for Chebulinic Acid and RA839

To characterize Keap1–inhibitor interactions, molecular dynamics (MD) simulations were performed on chebulinic acid (highest predicted binding affinity) and the reference compound 3899754 (selected as a reference due to its low IC_50_ value). The dynamic stability and structural changes of the complexes were evaluated using binding free energy (MM/GBSA), ligand efficiency (LE), root mean square deviation (RMSD) of the protein backbone, root mean square fluctuation (RMSF), and radius of gyration (Rg).

**Figure 4:**
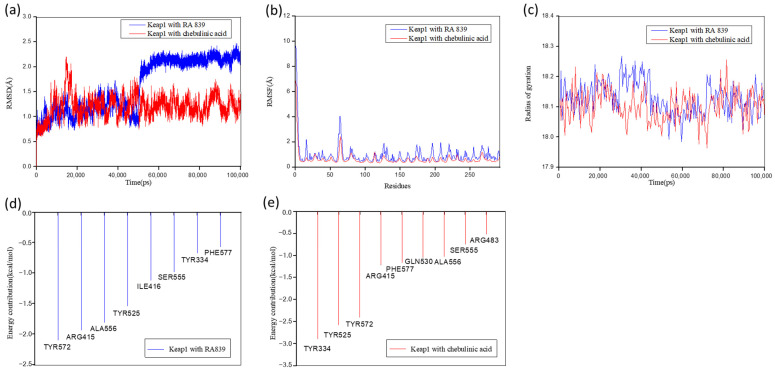
Molecular dynamics of chebulinic acid and reference (RA839) with Keap1 respective. (**a**) RMSD change profiles of two complex systems over 100 ns dynamic simulation. (**b**) RMSF values of binding site residues in two complex systems. (**c**) Gyration radius of two complex systems. (**d**) Residue energy contribution decomposition during the molecular dynamics simulation of Keap1 complexed with RA839. (**e**) Residue energy contribution decomposition during the molecular dynamics simulation of Keap1 complexed with chebulinic acid.

The RMSD values for both systems fluctuated around 2 Å, indicating stable binding (Figure 4a). The Keap1–RA839 complex showed an RMSD increase from 1 Å to 2.2 Å at ~50 ns before stabilizing, while the Keap1–chebulinic acid complex exhibited smaller deviations, ranging from 1 Å to 1.5 Å, suggesting superior conformational stability.

Residue flexibility was analyzed via RMSF over 100 ns of MD simulation (Figure 4b). The Keap1–chebulinic acid complex displayed lower overall flexibility compared to the reference Keap1–RA839 complex, implying stronger ligand binding stability at the active site. Both systems showed pronounced fluctuations in the Asn381–Ser390 loop region, which is distal to the binding pocket and may mediate the functional dynamics of Keap1.

Radius of gyration (Rg) analysis revealed that the Keap1–chebulinic acid complex had a smaller Rg value than the reference (Figure 4c), indicating a more compact and stable protein–ligand structure. This enhanced compactness correlated with chebulinic acid’s higher binding energy and suggested stronger inhibitory activity.

For the Keap1–chebulinic acid complex, residues Arg415, Tyr525, Ala556, and Tyr334 contributed most significantly to the binding energy (Figure 4d), confirming their role as key residues critical for ligand interaction. Notably, the reference complex (Keap1–RA839) exhibited similar key residues (Figure 4), further emphasizing the functional importance of these sites. These findings align with earlier docking results (Table 2), reinforcing the stability and specificity of chebulinic acid’s binding mode.

The average binding free energy of the Keap1–inhibitor complexes at the end of the 100 ns simulation period was calculated using the MM/GBSA method (Table 3). Chebulinic acid exhibited a strong binding affinity for Keap1, with a total binding energy of −22.17 ± 3.93 kcal/mol, though it was weaker than the reference inhibitor (−33.85 ± 5.14 kcal/mol for compound 3899754). Ligand efficiency (LE), which normalizes binding energy by molecular weight (Mw), was significantly lower for chebulinic acid due to its higher Mw. This highlights a common limitation of natural compounds: their larger size often reduces bioavailability. However, nano-delivery systems (e.g., lipid-based carriers or polymeric nanoparticles) could mitigate this issue by enhancing solubility and cellular uptake [30].

### 2.5. Validating the Predicted Results by In Vitro Experiment

#### Six Candidates Stimulated the Nrf2 Expression in HaCaT Cells

**Figure 5:**
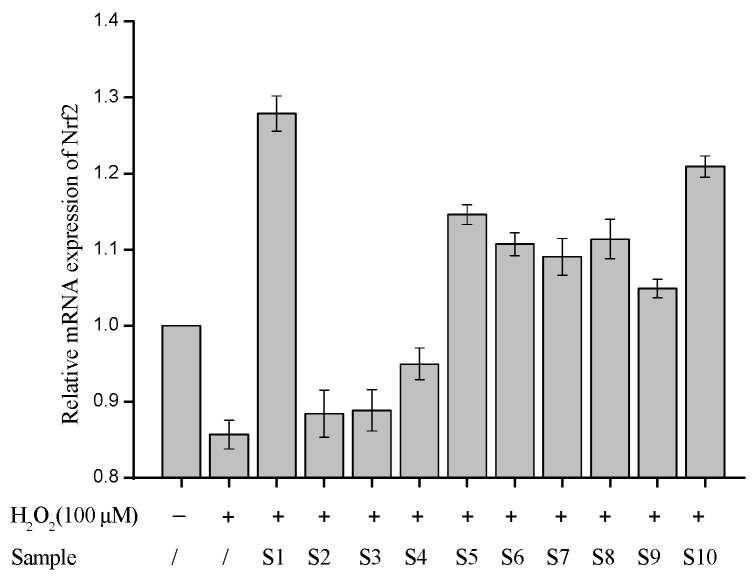
Relative mRNA expression of Nrf2 was quantified in HaCaT cells via RT−qPCR. Results are expressed as means ± SDs of three independent experiments. S1: chebulinic acid. S2: angoroside C. S3: angoroside C. S4: tubuloside B. S5: epmedin C. S6: rabdosiin. S7: 6‴-feruloylspinosin. S8: forsythiaside A. S9: cinnamtannin B-1. S10: RA 839.

To validate the virtual screening results, the nine candidates were tested in the model constructed in HaCaT cells that was stimulated with H_2_O_2_ to mimic the oxidative stress. Given the well-documented inverse correlation between NRF2 transcriptional activity and intracellular ROS levels [31,32], we employed NRF2 mRNA upregulation as a functional indicator to screen candidate inhibitors. From Figure 3, the mRNA expression of Nrf2 was significantly decreased under the oxidative stress of H_2_O_2_. S10 is a commercial agonist of Nrf2, as well as an inhibitor of Keap1-Nrf2. In this validation experiment, it was used as a positive control. It exhibited a promising ability to promote the mRNA expression of Nrf2, which indicated that this screening model is effective. Six of these nine compounds, including chebulinic acid, epmedin C, 6‴-feruloylspinosin, forsythiaside A, cinnamtannin B-1, and rabdosiin, exhibited a promising ability to promote the mRNA expression of Nrf2. The promotion rate ranged from 23% to 50%, compared with the control group (Figure 5).

Among the six candidates, chebulinic acid showed the highest increase in the mRNA expression of Nrf2 to combat oxidative stress. Additionally, the increasing rate was higher than that of the commercial inhibitor RA839. It was also consistent with the docking study and MD.

### 2.6. Penetration Study 

ADMET analysis (Table S2 and Table 3) revealed that chebulinic acid exhibited low bioavailability, limiting its direct application in the skincare industry. To address this, silk protein encapsulation was employed to enhance its penetration across the stratum corneum. Silk nanocarriers were optimized via high-speed homogenization, reducing their size from ~2000 nm to under 100 nm [33,34]. The smaller particle size enabled the efficient co-delivery of the chebulinic acid through the stratum corneum.

**Figure 6:**
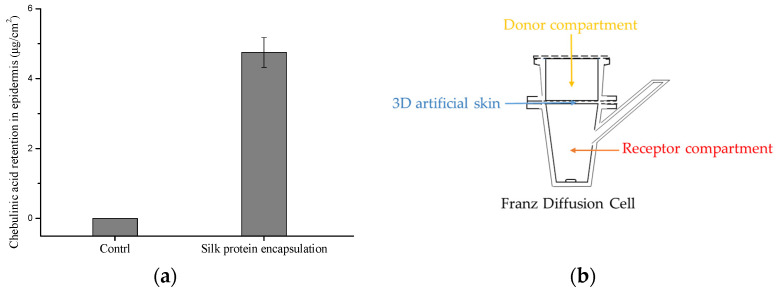
(**a**) Chebulinic acid recovery from the skin and receptor liquid after the application of silk protein encapsulation after 24 h (mean ± SD, *n* = 3); (**b**) Franz diffusion cell.

Quantitative UHPLC-MS analysis demonstrated a chebulinic acid loading capacity of 2000 mg/L in the silk fibroin-based drug delivery system (Supplementary Information, Figure S2). As shown in Figure 6, unencapsulated chebulinic acid failed to permeate the stratum corneum, whereas the encapsulated formulation significantly improved bioavailability. This is attributed to chebulinic acid’s high molecular weight, which hinders transdermal delivery.

This indicated that it could be applied as an inhibitor of Keap1-Nrf2 to combat oxidative stress in the cosmetics industry. It also showed that a virtual screening model with delivered technology could be an effective solution to screen active compounds in the skincare industry.

## 3. Materials and Methods

### 3.1. Experimental Procedure

**Figure 7:**
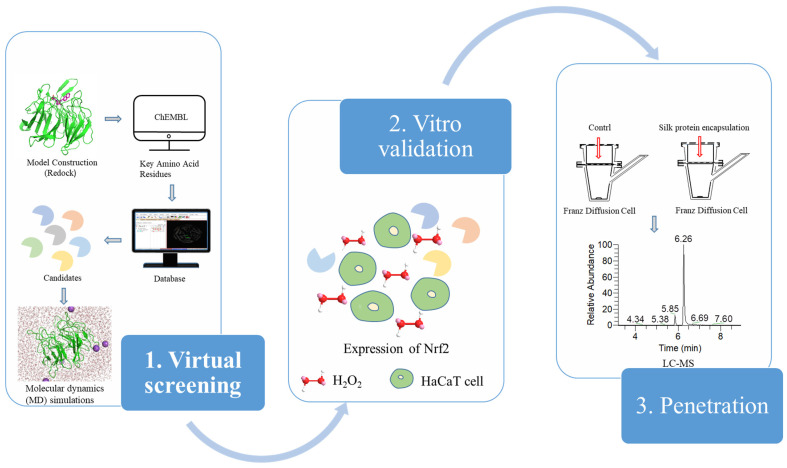
Flow chart of the experimental procedure. The blue directional arrows explicitly define the procedural workflow. In the ball-and-stick molecular representation of hydrogen peroxide (H_2_O_2_), oxygen atoms are represented by red spheres, whereas the smaller hydrogen atoms are shown as white spheres, with covalent bonds illustrated by interconnecting rods. Circles from which a quarter has been excised represent candidate compounds derived from the virtual screening and diverse colors of circles are utilized to signify different classes of compounds. The HaCaT cells are shown as irregular green circles, each containing a small pink circle within.

Figure 7 shows the workflow utilized in this report.

### 3.2. Materials

#### 3.2.1. Reagents and Materials

Acetonitrile (ACN), ethanol (EtOH), and methanol (MeOH) of the HPLC grade were purchased from Sigma Aldrich (Milan, Italy). Formic acid (FA), also of the HPLC grade, was obtained from Shanghai Anpel Experimental Technology Co., Ltd. (Shanghai, China). Sodium chloride (NaCl) was purchased from Sinopharm Chemical Reagent Co., Ltd. (Shanghai, China). Milli-Q water was obtained in-house using a Millipore system (Bedford, MA, USA). Chebulinic acid, tubuloside B, angoroside C, epmedin C, sennoside B, cinnamtannin B-1, 6‴-Feruloylspinosin, forsythiaside A, and rabdosiin were purchased from Topscience Co., Ltd. (Shanghai, China). Silk protein (BNA-F-F, containing 0.5% fibroin, 5% pentylene glycol, and 94.5% water) was purchased from Sinate Biotechnology Co. (Suzhou, China).

#### 3.2.2. Software for Molecular Docking and Visualization

AutoDock Vina version 4.2.6 was employed for docking simulations [35]. The interactions between the ligand–protein complexes were visualized using PyMol version 1.1.7 and the PLIP online server “https://plip-tool.biotec.tu-dresden.de/ (accessed on 18 March 2024)”.

### 3.3. Model Construction for Molecular Docking 

#### 3.3.1. Protein Preparation

Three-dimensional coordinates of Keap1 (PDB code 8IXS) were retrieved from the Protein Data Bank “http://www.rcsb.org (accessed on 12 March 2024)” [11]. The PDB file was examined for missing side chains and subsequently processed using AutoDockTools (ADT version 1.5.6) to prepare the PDBQT file. During this process, water molecules and ions were removed, only polar hydrogens were retained, and Gasteiger charges were computed for the protein atoms using ADT.

#### 3.3.2. Ligands Preparation

A database of natural compounds containing over 16,000 entries from TSbitochem “www.tsbiochem.com (accessed on 16 March 2024)” was utilized for virtual screening. All the molecules in the database were automatically converted to the PDBQT format using OpenBabel (version 3.1.1).

#### 3.3.3. Docking Procedure 

The 3D structures of the target proteins, provided in .pdb format, were obtained from the Protein Data Bank (PDB) database “http://www.rcsb.org (accessed on 16 March 2024)”. AutoDock Vina was utilized for docking to ascertain the optimal conformation. The docking pocket was delineated based on the original ligand’s position within the target protein complex. Following the docking process, the interactions between the active compounds and target proteins were analyzed using PyMOL, which also generated the complex .pdb file for further investigation of the interactions between the ligand and the protein via PLIP. The key amino acids essential for the screening model were identified based on the docking data of ten inhibitors from ChEMBL.

### 3.4. ADMET Study for the Candidate Inhibitors

The “in silico ADMET (absorption, distribution, metabolism, excretion, and toxicity) screening” of the selected inhibitors was conducted on the “PKCSM online server” [36].

### 3.5. Molecular Dynamics (MD) Simulations

Molecular dynamics (MD) simulations (100 ns, AMBER force field, 300 K) were performed to analyze the conformational stability of the top-ranked Keap1–chebulinic acid complexes and to quantify flexibility in the Keap1 binding pocket. The simulations were conducted on the Yinfo Cloud Computing Platform “https://cloud.yinfotek.com/ (accessed on 18 March 2024)” using the AmberTools(version 20.0). The AMBER ff19SB force field [37] and generalized AMBER force field (GAFF) [38] were applied to Keap1 and chebulinic acid, respectively.

The system setup involved the solvation of the docked complex in an OPC water model within an orthorhombic box (10 Å buffer distance) and neutralization with 0.15 M NaCl. The simulation protocol comprised four stages: minimization: first stage: 20,000 steps of the steepest descent minimization with restraints (10 kcal·mol^−1^·Å^−2^) on the complex; second stage: 20,000 steps of conjugate gradient minimization with restraints (10 kcal·mol^−1^·Å^−2^) on the protein backbone; And third stage: 20,000 steps of conjugate gradient minimization without restraints. Heating: A 20 ps NVT ensemble equilibration at 300 K employing Langevin dynamics (collision frequency = 1 ps^−1^) with restraints (10 kcal·mol^−1^·Å^−2^) on the complex. Equilibration: 200 ps NPT ensemble equilibration at 1 atm pressure with restraints (10 kcal·mol^−1^·Å^−2^) on the protein backbone and 1 ns NVT ensemble equilibration without restraints. Production: A 100 ns unrestrained MD simulation in the NVT ensemble. Post-simulation analyses included root mean square deviation (RMSD), root mean square fluctuation (RMSF), radius of gyration (Rg), and protein–ligand interaction contacts, performed using the CPPTRAJ module in AmberTools.

### 3.6. Cell-Based Assays

#### 3.6.1. Cell Culture and H_2_O_2_ Treatment

Human epithelial keratinocyte (HaCaT) cells were purchased from Sunncell Biotechnology Co., Ltd. (Wuhan, China). HaCaT cells were maintained in DMEM (Gibco #11965092) under standard culture conditions (37 °C, 5% CO_2_), supplemented with 10% fetal bovine serum (FBS; Gibco, Cat. No. 10091148) and 1% penicillin/streptomycin (P/S; Gibco, Cat. No. 15140122). Subsequently, the HaCaT cells were co-treated with candidate compounds and H_2_O_2_ (100 µM) and incubated for 24 h.

#### 3.6.2. RNA Extraction and RT-qPCR Testing 

Total RNA was extracted using the RNeasy Mini Kit (Qiagen, Hilden, Germany) following the manufacturer’s instructions. The extracted RNA was quantified using Synergy H1 (BioTek, MA, USA). Subsequently, the RNA was reverse-transcribed into complementary DNA (cDNA) using the PrimeScript™ RT reagent kit (Takara, Shiga, Japan). The expression of Nrf2 was assessed using SYBR Premix Ex Taq II (Takara, Shiga, Japan) with the forward primer 5′-ACAATGAGGTTTCTTCGGCTAC-3′ and the reverse primer 5′-CGTCTAAATCAACAGGGGCTAC-3′. All quantitative PCR (qPCR) analyses were conducted with the QuantStudio 3 Real-Time PCR Instrument (Thermo Scientific, MA, USA). The relative expression level of the target gene was calculated using the ddCt method, with GAPDH serving as the internal control.

### 3.7. Bioavailability Study for the Candidate Inhibitor

#### 3.7.1. Silk Protein Encapsulation Technology

In order to be applicable in cosmetics, the active molecules should be penetrated into the stratum corneum of the skin. Actives from nature extract have the drawback of not being able to easily penetrate the stratum corneum of the skin. In this situation, actives-delivered technologies could be applied to help the actives penetrate into the stratum corneum, including nanocream, lipsome, and so on. In this experiment, silk protein encapsulation technology was applied [39]. The selected candidate substances with silk protein encapsulation were compared to those without silk protein encapsulation.

A mixture of 5 g of diethylene glycol monoethyl ether (DEGME) and 5.4 g of pure water was combined with 0.1 g of chebulinic acid powder to form phase A. This phase was stirred and dissolved until it became transparent and uniform. Concurrently, 14.5 g of butanediol and 25 g of BNA-F-F were mixed uniformly to create phase B. Subsequently, phase A was added dropwise to phase B at room temperature while maintaining constant stirring for 0.5 h to ensure the complete loading of chebulinic acid onto the silk fibroin. Finally, after 5 min of homogenization at 7000 rpm through T 25 digital ULTRA-TURRAX^®^ (IKA, Staufen im Breisgau, Germany), the silk fibroin-encapsulated chebulinic acid was obtained.

#### 3.7.2. Franz Diffusion Cell (FDC)

Franz diffusion cells (FDC; Phoenix DB-6, Hanson) with a 0.33 cm^2^ diffusion surface and a 7.0 mL receptor volume were employed. Reconstructed human epidermis (RHE) samples were pre-conditioned with isotonic phosphate-buffered saline (PBS, pH 7.4) and positioned between the donor and receptor chambers with the stratum corneum layer oriented upward. The receptor compartment received 7.0 mL of PBS (37 °C) degassed via sonication to eliminate microbubbles at the membrane–solution interface.

The experimental design comprised the test group: 0.2 mL of chebulinic acid (1% *w*/*v*) encapsulated in silk fibroin matrix (*n* = 3) and the control group: 0.2 mL of free chebulinic acid solution (1% *w*/*v*, *n* = 3).

The system was maintained at 37 ± 0.5 °C via a thermostatically controlled water jacket. Receptor medium was continuously agitated at 600 rpm using a Teflon-coated magnetic stir bar (Ø5 × 10 mm) to maintain sink conditions. Independent triplicate experiments were conducted with fresh RHE specimens for each trial.

At 24-h intervals, cells were harvested from the temperature-controlled incubator, and the collected samples (*n* = 3 per time point) were analyzed in triplicate using ultra-high-performance liquid chromatography (UHPLC) to quantify chebulinic acid levels.

#### 3.7.3. UHPLC-MS

The chromatographic separation was performed on a Welch Xtimate UHPLC C18 column (2.1 × 100 mm, 1.8 µm) using a mobile phase system consisting of (A) 0.1% formic acid in water and (B) 0.1% formic acid in methanol. The separation was achieved with a flow rate of 300 μL/min under the following gradient program: Initial composition was maintained at 10% B for 2 min, followed by a linear gradient from 10% to 90% B over 4 min. The 90% B condition was held for 2 min before returning to initial conditions through a 2 min linear gradient from 90% to 10% B. The column was subsequently re-equilibrated with 10% B for an additional 2 min to complete the 12 min analytical cycle.

Chebulinic acid was analyzed using a Q-Exactive Hybrid Quadrupole-Orbitrap mass spectrometer (Thermo Fisher Scientific, Waltham, MA, USA) in ESI negative mode. Full-scan spectra (resolution: 70,000 at *m*/*z* 200) and targeted MS/MS (collision energy: 30 eV) were collected over m/z 100–1500 Da. Other parameters followed established protocols [40].

## 4. Conclusions

A virtual screening model for the non-covalent inhibition of the Keap1-Nrf2 interaction was developed using molecular docking. Selection criteria were binding energy < −9 kcal/mol and interactions with 4–5 key residues, and the model was validated against seven commercial inhibitors.

Nine natural compounds, including *chebulinic acid*, *tubuloside B*, and *angoroside C*, were identified. MD simulations and cell-based assays (qPCR in HaCaT keratinocytes) confirmed that six compounds upregulated Nrf2 mRNA by 23–50%, and *chebulinic acid* had the highest increase. Silk fibroin encapsulation enhanced *chebulinic acid’s* transdermal delivery via sub-100 nm nanoparticles, overcoming stratum corneum barriers.

This work demonstrated that integrating virtual screening with delivery technologies can identify potent, naturally derived Keap1-Nrf2 inhibitors for anti-aging skincare. However, cost constraints limited the analysis of some inhibitors; experimental studying to validate the interaction between Keap1 and the inhibitor, such as surface plasmon resonance (SPR), long-time MD simulation to probe rare events, and clinical studies are needed to validate safety/efficacy. Future work will prioritize translational research to bridge this gap.

## Figures and Tables

**Table 1:** Residuals involved in the interaction between seven commercial inhibitors and Keap1.

ChEMBL ID	IC50 (nM)	Binding Energy (kcal/mol)	Residual
3899754	0.14	−11.6	Phe557, Tyr334, Ser363, Arg415, ALa556, Ser508, Phe478, Arg483
3601212	14.40	−9.3	Arg483, Tyr525, Ser555, Ala556, Arg415, Tyr334, Ser363, Asn382
4544116	15.80	−10.4	Arg483, Tyr525, Gln530, Phe478, Asn414, Ala556, Ser602, Asn382
4757197	48.00	−9.4	Tyr572, Gln530, Ile559, Val512, Tyr525, Arg415, Arg483, Ile461, Phe478
4174651	60.00	−9.7	Asn382, Asn414, Ser363, Arg415, Tyr334, Ser602, Ala556, Ser555, Tyr525, Ser508, Arg483
4646536	73.00	−9.2	Ser508, Tyr525, Ser555, Ala556, Tyr334, Ser363, Arg415
4762197	120.00	−10.4	Ser363, Asn382, Tyr334, Arg415, Ala556, Phe577, Gln530

**Table 2:** Binding energy and interactive residuals involved in the interaction between nine inhibitors and Keap1.

Compound	Binding Energy (kcal/mol)	Residuals	
		Hydrogen bonding	Hydrophobic interactions
Chebulinic acid	−10.4	Ser363, Asn382, Asn414, Arg415, Ile461, Arg483, Tyr525, Gln530, Ser602	Tyr334, Tyr525, Ala556
Angoroside C	−10.3	Arg415, Arg483, Tyr525, Ser555, Ser602	Tyr334, Arg415, Ala556
Sennoside B	−10.1	Ser363, Asn382, Val418, Ile461, Val465, Ser508, Gly509, Val512, Tyr525, Gln530, Leu557, Ser602	Tyr334, Arg415, Phe478, Tyr525, Ala556
Tubuloside B	−9.9	Ser363, Asn382, Ile416, Val463, Arg483, Ser508, Gln530, Ser602, Tyr334, Arg415	Tyr525, Ala556
Epmedin C	−9.8	Arg483, Tyr525, Gln530, Ser555, Leu557	Tyr334, Arg415, Ala556, Tyr572
Rabdosiin	−9.7	Ser363, Asn382, Gly462, Arg483, Ser508, Tyr525, Gln530, Tyr572, Ser602	Tyr334, Arg415, Tyr525, Ala556, Tyr572, Phe577
6‴-Feruloylspinosin	−9.3	Ser363, Asn382, Asn414, Arg415, Arg483, Ser508, Tyr525, Gln530, Ser555, Ser602	Tyr334, Arg415, Tyr525, Ala556
Forsythiaside A	−9.2	Ser363, Gly364, Asn382, Arg415, Arg483, Ser508, Ala510, Gln530, Ala556	Tyr334, Tyr525, Ala556
Cinnamtannin B-1	−9.2	Tyr334, Arg415, Arg483, Gly509, Tyr525, Gln530, Tyr572	Tyr334, Tyr525, Ala556

**Table 3:** Binding energy and LE comparsion for 2 complex systems.

Item	Complex of Chebulinic Acid	Complex of RA839
ΔG total	−22.17 ± 3.93	−33.85 ± 5.14
LE	0.32	1.05

The binding free energy (ΔG total) is defined as the sum of the intrinsic binding free energy between the protein and ligand in the absence of solvent (vacuum) and the solvation free energy contributions. ΔG total = ΔG vacuum + ΔG solv.

## Data Availability

Data are contained within the article.

## Supplementary Materials

The following supporting information can be downloaded at: https://www.mdpi.com/article/10.3390/molecules30081815/s1, Figure S1: Binding of chebulinic acid (a), tubuloside B (b), angoroside C (c), epmedin C (d), sennoside B (e), cinnamtannin B-1 (f), 6‴-Feruloylspinosin (g), forsythiaside A (h), and rabdosiin (i) into the active site of Keap1; Figure S2: Extracted ion chromatograms of chebulinic acid; Table S1: Compounds for virtual screening and their binding energies with the Keap1 protein. Table S2. The ADMET profile of RA839 and nine candidate compounds.
