# Supplementary material for: Virtual Screening of Kelch-like ECH-Associated Protein 1-Nuclear Factor Erythroid 2-Related Factor 2 (Keap1-Nrf2) Inhibitors and In Vitro Validation
Source: Molecules. 2025 Apr 17;30(8):1815. doi: 10.3390/molecules30081815 (PMC12029559; doi:10.3390/molecules30081815)
Supplement: Supplementary file 1 [file molecules-30-01815-s001.zip › Supplementary Materials.pdf]

Supplementary Materials: The following supporting information can be downloaded at: www.mdpi.com/xxx/s1, Figure S1: Binding of chebulinic acid (a), tubuloside B (b), angoroside C (c), epmedin C (d), sennoside B (e), cinnamtannin B-1 (f), 6‴-Feruloylspinosin (g), forsythiaside A (h), and rabdosiin (i) into the active site of Keap1; Figure S2: Extracted ion chromatograms of chebulinic acid; Table S1: Compounds for virtual screening and their binding energies with the Keap1 protein. Table S2. The ADMET profile of RA839 and nine candidate compounds

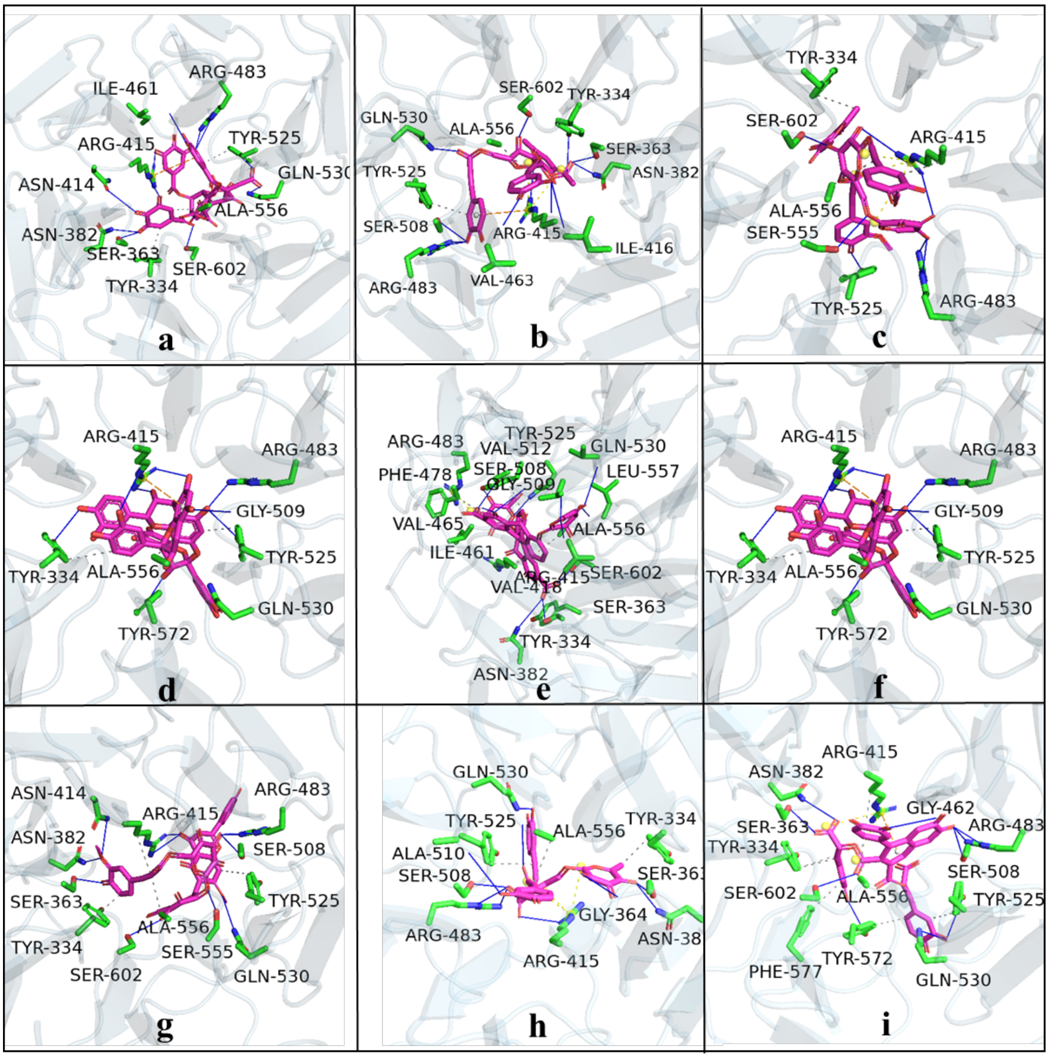

Figure S1. Binding of chebulinic acid (a), tubuloside B (b), angoroside C (c), epmedin C (d), sennoside B (e), cinnamtannin B-1 (f), 6‴-Feruloylspinosin (g), forsythiaside A (h), and rabdosiin (i) into the active site of Keap1.

https://doi.org/10.3390/xxxxx

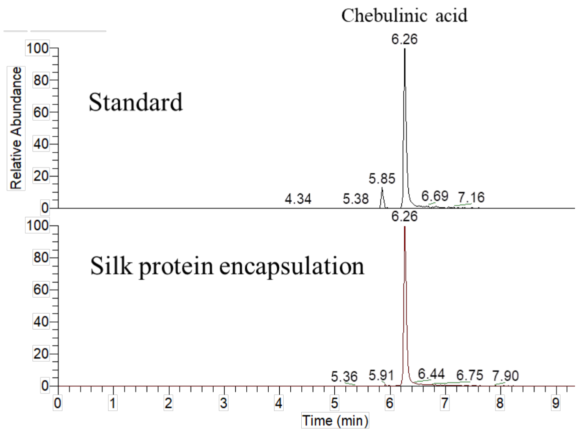

Figure S2. Extracted ion chromatograms of chebulinic acid. The top panel was the extracted ion chromatograms of the raw file from a 10 mg/L chebulinic acid, whereas the bottom panel was extracted ion chromatograms of chebulinic acid from silk protein encapsulation sample, which was diluted 10 times with 10 % ethanol.
